# Managing Follicular Lymphoma in the Elderly Population

**DOI:** 10.1155/2023/1038934

**Published:** 2023-01-05

**Authors:** Jiao Jie Cherie Tan, Yuen Lei Sze, Clarice Choong Shi Hui

**Affiliations:** ^1^Division of Haematology, Department of Medicine, National University Hospital, Singapore; ^2^Division of Geriatric Medicine, Department of Medicine, Ng Teng Fong General Hospital, Singapore; ^3^Division of Haematology, Department of Haematology-Oncology, National University Cancer Institute, Singapore

## Abstract

Follicular lymphoma (FL) is one of the most commonly diagnosed types of indolent non-Hodgkin lymphoma (NHL). The median age of diagnosis for FL is 65 years old. Although the median life expectancy after diagnosis is approximately 10 years, the incurable disease has a high risk of transformation. This case report focuses on an 80-year-old patient diagnosed with low-grade follicular lymphoma which subsequently transformed leading to the patient's eventual demise as the patient took on the palliative intent. This case report aims to highlight the importance of clinical markers or prognostic factors to identify patients, specifically the elderly population who are at risk of transformation to aggressive forms when their FL remains at stage I-II phases. Currently, elderly patients with FL tend to be quickly dismissed with curative intent with chemotherapy, given their age and comorbidities, despite forming the majority of the population with follicular lymphoma. Age more than 60 years old has been shown to be one of the most powerful yet poor prognostic features in follicular lymphoma international prognostic index (FLIPI)—the main scoring system used for FL. Hence, further studies are required to look into the tailoring treatment for elderly patients with follicular lymphoma after risk stratifying them with appropriate clinical and prognostic markers.

## 1. Introduction

Follicular lymphoma accounts for 20–30% of all newly diagnosed non-Hodgkin's lymphoma [1] and is typically diagnosed in the elderly. Despite the advancement of the treatment, FL remains an incurable disease. 15–28% transform into an aggressive phenotype, typically the diffuse large B cell lymphoma [2].

The treatment of follicular lymphoma depends on the stage of the disease. The staging of lymphoma preliminarily includes either CT scan or PET-CT scan, bone marrow aspirate, and biopsy. Subsequently, the staging is performed based on the Ann Arbor classification, which classifies patients into either curative or palliative tracks.

As a result of standardised practices, scoring systems such as the follicular lymphoma international prognostic index (FLIPI) were introduced in 2004 and subsequently revised to FLIP2 after the introduction of rituximab. The overall survival (OS) of patients with FL has improved significantly since the introduction of rituximab [3]. However, as these scores are not age adjusted, the elderly FL population tends to have at least one risk factor including age more than 60 years old.

80% of patients experience long‐term disease control following standard chemoimmunotherapy combinations or even without the need of treatment. However, an estimated 16% have early progression and meet the treatment initiation criteria within 1 year of diagnosis [4], with probably a significantly shorter survival duration (median, 48 months) [5]. There is also strong evidence of poor outcomes if progression of the disease is within 24 months of diagnosis after first-line chemoimmunotherapy (POD24), with up to 20–50% risk of death in 5 years [6, 7].

We know that the elderly population is more vulnerable to side effects of immune-suppressive treatment including neurotoxicity, hepatotoxicity, and electrolyte derangement. It is also known that the elderly's DNA damage repair mechanism and immune response differ from the younger general population [8].

However, as FL-directed therapy improves, patients diagnosed with FL at 80 years old were associated with improved survival [9].

Studies have shown that with appropriate chemotherapy of reduced intensity, these elderly patients with haematological malignancies can continue to proceed with a good quality of life [10].

Hence, this case report aims to highlight the importance of developing prognostic markers specifically targeted at the elderly population with follicular lymphoma. This is the key to better tailor treatment options for the elderly population, improving overall survival and quality of life.

## 2. Case Presentation

An 81-year-old Chinese male presented to the emergency department with fever, hypoactive delirium, and functional decline in May 2021. He was known to have chondrosarcoma of the right hip with internal fixation to the right hip more than 20 years ago. Other background history included moderate stage of Alzheimer's dementia, peptic ulcer disease, type 2 diabetes mellitus, hypertension, ischaemic heart disease, and heart failure with preserved ejection fraction.

He was diagnosed with follicular lymphoma grade 1-2 in November 2019. The lymphoma was first identified as a loculated mass on CT TAP. After exploratory laparotomy and small bowel resection in February 2020, a repeated CT scan at the haematology clinic showed no remnant or suspicious soft tissue focus. The patient was asymptomatic, and hence, no further chemotherapy or treatment was required.

He represented in May 2021 with sepsis and functional decline. Despite multiple courses of antibiotics to treat pneumonia and urinary tract infection, he continued to suffer from persistent fever due to his lymphoma. A CT scan showed a 9.4 × 4.0 cm right pelvic mass, and MRI pelvis showed that the right pelvis mass had encased on the right internal iliac vessels and right S1 and S2 nerve roots. There was disseminated marrow infiltration consistent with metastasis. Interventional radiographical biopsy of the right pelvic mass revealed low-grade follicular lymphoma (WHO grade 1 or 2). Immunochemistry from initial small bowel resection showed that the small lymphoid cells were positive for CD20, CD10, BCL2, and BCL6 (weak expression). The centroblast count is less than 15 per 0.159 mm^2^. On immunohistochemical staining, a few small CD 20+ B cells and some small CD3+ T cells are seen with large atypical B cells or aggregates of B cells are noted. The tissue sampled initially represented grades 1-2 follicular lymphoma.

He was referred to haematology, and the recommended treatment was for palliative radiotherapy to the right abdomen and pelvis, which was completed in August 2021. He remained chairbound and basic-activity of daily living (ADL)-dependent despite a trial of rehabilitation.

He was readmitted in December 2021 for hypoactive delirium and was given a course of antibiotics for the urinary tract infection initially. However, his malignant fever persisted. He was also found to have right lower limb below-knee deep vein thrombosis and was started on rivaroxaban. Computer tomography of his thorax, abdomen, and pelvis showed new small bibasal pleural effusions and new moderate ascites. MRI pelvis showed Ill-defined infiltrative lesions noted along the right lateral pelvic wall encasing the right lateral pelvic wall and the neurovascular bundles and lucent and sclerotic areas in the pelvis and lumbar spine possibly due to diffuse marrow infiltration. MRI of the brain showed scattered foci of marrow replacing lesions in the skull, suspicious of lymphomatous deposits. Pleural and ascites studies showed lymphocytic exudative effusion. Histology showed the presence of atypical lymphocytic cells suggestive of lymphoma cells in both fluids. On immunostaining of the peritoneal fluid, the malignant cells were diffusely positive with CD20 and CD3 highlighted scattered reactive small lymphoid cells, and CD71 shows some weak nonscattered mononuclear cells.

He developed complications from his lymphoma including new bilateral pleural effusions, ascites, new infiltrative lesions in his right pelvis, and brain involvement as his lymphoma transformed and progressed rapidly.

His family was updated on his grave prognosis in view of the diagnosis of stage 4 follicular lymphoma. A family conference was held, and the goals of care and the treatment were rediscussed. The family agreed with the plan to support the patient with palliative management and for withdrawal of antibiotics. The patient passed away 2 days afterwards.

## 3. Discussion

This case report demonstrates the importance of identifying groups of elderly patients who are at an early stage of follicular lymphoma with high risk of transformation. With the advancement of medical treatment and investigation in treating patients, age alone should not disqualify patients from the standard treatment or monitoring.

Currently, the initiation and selection of the treatment from various guidelines are based on symptoms, staging of the disease according to the Ann Arbor classification system, histological grading, and tumour burden assessment with GELF, BNLI, or NCCN criteria [11]. The watch-and-wait (W/W) approach is generally implemented for patients with asymptomatic, low tumour burden, stage II–IV low-grade FL until the occurrence of symptoms or signs of advancing lymphoma (e.g., B symptoms, bulky disease, organ involvement, ascites or pleural effusion, rapid progression, or bone marrow infiltration with cytopenias) [12]. Studies to-date both in the pre and postrituximab era have not shown an OS benefit from the immediate treatment in such low-risk patients [13, 14].

The tumour immune microenvironment, host immune response, and immune checkpoint are relevant in the pathogenesis of follicular lymphoma. We know that high percentages of FOXP3 regulative T lymphocytes (Tregs) and high programmed cell death protein 1 (PD-1) follicular T lymphocytes (TFH cells) are associated with an overall good survival of the follicular lymphoma patients. Yet at the same time, there is a higher risk of transformation to diffuse large B cell lymphoma (DLBCL). [15].

On the other hand, there is also an association with high frequencies of cells mainly macrophages and follicular lymphoma B centroblast associated with poor overall survival. Several immune microenvironment markers have been identified including CD163, CSF1R, FOXP3, PDCD1, and IL10. Interestingly, these sets of genes were correlated with other known follicular lymphoma prognostic markers including international prognostic index (IPI) score [16].

The transformation of FL to diffuse large B cell lymphoma is defined as pathologically demonstrated and clonally confirmed progression of FL grade 1–2 and 3a to DLBCL or less commonly the intermediate gray zone category of unclassifiable B cell lymphoma. Thus, diagnostic biopsy from the composite histology finding is imperative to imply (although it does not confirm) early transformation. [17].

Moving back to the case, immunochemistry from the initial small bowel resection represented grade 1–2 follicular lymphoma with weak expressions of molecular markers capable of the high-grade transformation.

Unfortunately, during the latest admission when he was admitted for infections and deteriorated rapidly, the immunostaining of his peritoneal fluid showed malignant cells that were diffusely positive with CD20, CD3 highlighted scattered reactive small lymphoid cells, and CD71 shows some weak nonscattering of mononuclear cells. With the limited information from immunostaining from the peritoneal fluid, the presence of CD20 could be a possible indicator of high risk of transformation. The neoplastic cells of FL typically express CD 19, CD20, CD22, and CD 79a and show surface Ig expression—serum immunoglobulin of IgM ± IgD, IgG, or rarely IgA. Most cases are also positive for BCL2, BCL6, and CD10 [18].

In addition to biopsy proven evidence of the progression to an aggressive lymphoid malignancy, an unequivocal definition of transformation requires demonstration of a clonal relationship between original FL and subsequent neoplasm. This can be established by molecular techniques demonstrating the use of immunoglobulin gene comprising variable, diversity, and joining segment that shares a backbone of common somatic mutations, thus allowing inference of a common progenitor cell. While transformed DLBCL frequently maintains the immunophenotype of the preceding FL, antigenic drift may occur during the transformation process and thus the loss or gain of antigenic markers does not preclude a clonal relationship between FL and subsequent transformed histology. This makes distinction to DLBCL from the FL complex [19]. Furthermore, in the context of a geriatric haematology population, the indication to treat this group of patients becomes challenging.

There is also a lack of evidence that the W/W approach and delaying FL-directed therapy increase the survival. Hence, the debate remains whether the treatment should be dismissed or delayed. This is especially more important for the elderly who always have a short runway for starting the therapy. The elderly population is at a high risk of the progress into frailty once they are symptomatic. Generally, for the W/W strategy, patients need to understand that delaying the therapy may have an adverse impact on their survival; and they are supposed to be followed up closely (e.g., every 3 months) during the first year of surveillance, before moving to less intensive observation since FL is a cancer and occasionally progress rapidly [20]. The annual CT scanning may be appropriate to offer and reassure the patient with isolated abdominal disease. The use of PET to monitor FL during the observation period is not recommended [21].

Our patient defaulted follow-up because of his belief and mindset that FL generally has a good prognosis. He was not started on chemoimmunotherapy after the diagnosis because he was considered as asymptomatic with limited disease and low tumour burden. By the time he presented again to the haematologist one year later with B symptoms, bulky disease, as well as high tumour burden, he was deemed to be too frail and not fit for chemoimmunotherapy in view of his recurrent sepsis as well as multiple poor prognostic factors (cognitive impairment with poor function, multiple comorbidities, high C-reactive protein, and low albumin level) [22]. His early and rapid symptomatic progression is not related to any histological transformation. He continued to deteriorate rapidly with the development of malignant pleural effusion and ascites possibly from peritoneal lymphomatosis, and subsequently palliative management was the only option left for him.

Identifying differences in patients with early versus late or no progression is a critical step moving forward in understanding outcomes and appropriate patient selection for the W/W strategy. Current studies on various clinical and prognostic indices such as IPI, ILI, FLIPI, FLIPI2, and PRIMA-PI have been validated for their ability to predict progression-free, response to treatment, early relapse (POD24), histological transformation (HT), or OS in patients receiving initial chemoimmunotherapy [23, 24], but they are unable to provide aid in deciding when to initiate the therapy or identify the subgroup of high-risk patients at the time of diagnosis [25]. The use of FDG-PET/CT is important for staging and has greater sensitivity compared to the standard CT in detecting additional nodal and extranodal sites of disease [26] and hence altering management after upstaging of FL by the scan [27, 28]. Recent identification of the baseline total metabolic tumour volume (TMTV) correlated with PET imaging represents an early predictor of high-risk patients and assists with risk-adapted approaches to the treatment [29]. There is also controversial evidence that baseline and end-of-induction PET status such as whole-body maximum standardised uptake (SUVmax) was a significant predictor of survival in patients receiving rituximab [30, 31]. However, both TMTV and SUVmax are prognostic tools for predicting the outcome in patients receiving chemoimmunotherapy.

## 4. Conclusion

We hope that in the future, new indices such as PET imaging modalities will be validated to stratify patients at the time of diagnosis more accurately. Risk stratification in terms of patients who are truly indolent course and allow W/W approach, versus the subpopulation of patients harbouring aggressive diseases like our patients here, who may benefit from the early FL-directed therapy. Hence, this case report hopes to highlight the importance of identifying clinical markers or prognosticators which can be used to identify the patients specifically the elderly patients who are at a high risk of transformation to increase surveillance and reduce the risk of disease progression for these patients.

## Data Availability

Data sharing is not applicable to this article as no datasets were generated or analysed during the current study.

## References

[1] Hanel W., Epperla N. (2021). Evolving therapeutic landscape in follicular lymphoma: a look at emerging and investigational therapies. *Journal of Hematology & Oncology*.

[2] Lossos I. S., Gascoyne R. D. (2011). Transformation of follicular lymphoma. *Best Practice & Research Clinical Haematology*.

[3] Bitansky G., Vasilev E., Zlotnick M. (2019). Progression of follicular lymphoma within 24 Months of first treatment (POD -24) as a predictor of overall survival - a single center retrospective analysis. *Blood*.

[4] Vose J. M., Armitage J. O., Weisenburger D. D. (1988). The importance of age in survival of patients treated with chemotherapy for aggressive non-Hodgkin’s lymphoma. *Journal of Clinical Oncology*.

[5] Castellino A., Vitolo U., Nicolosi M., Botto B., Boccomini C., Santambrogio E. (2016). Follicular lymphoma: the management of elderly patient. *Mediterranean Journal of Hematology and Infectious Diseases*.

[6] Khurana A., Mwangi R., Ansell S. M. (2021). Patterns of therapy initiation during the first decade for patients with follicular lymphoma who were observed at diagnosis in the rituximab era. *Blood Cancer Journal*.

[7] Brice P., Bastion Y., Lepage E. (1997). Comparison in low-tumor-burden follicular lymphomas between an initial no-treatment policy, prednimustine, or interferon alfa: a randomized study from the Groupe d’Etude des Lymphomes Folliculaires. Groupe d’Etude des Lymphomes de l’Adulte. *Journal of Clinical Oncology*.

[8] Fisher R. I., LeBlanc M., Press O. W., Maloney D. G., Unger J. M., Miller T. P. (2005). New treatment options have changed the survival of patients with follicular lymphoma. *Journal of Clinical Oncology*.

[9] Maurer M. J., Bachy E., Ghesquières H. (2016). Early event status informs subsequent outcome in newly diagnosed follicular lymphoma. *American Journal of Hematology*.

[10] Albarmawi H., Onukwugha E., Keating K. N., Appukkuttan S., Yared J. (2020). Survival benefit associated with treating follicular lymphoma in patients 80 years or older. *Journal of Geriatric Oncology*.

[11] Cartron G., Trotman J. (2022). Time for an individualized approach to first-line management of follicular lymphoma. *Haematologica*.

[12] Kuruvilla J., Assouline S., Hodgson D. (2015). A Canadian evidence-based guideline for the first-line treatment of follicular lymphoma: joint consensus of the Lymphoma Canada Scientific Advisory Board. *Clinical Lymphoma, Myeloma & Leukemia*.

[13] Ardeshna K. M., Qian W., Smith P. (2014). Rituximab versus a watch-and-wait approach in patients with advanced-stage, asymptomatic, non-bulky follicular lymphoma: an open-label randomised phase 3 trial. *The Lancet Oncology*.

[14] El-Galaly T. C., Bilgrau A. E., de Nully Brown P. (2015). A population-based study of prognosis in advanced stage follicular lymphoma managed by watch and wait. *British Journal of Haematology*.

[15] de Jong D., Koster A., Hagenbeek A. (2009). Impact of the tumor microenvironment on prognosis in follicular lymphoma is dependent on specific treatment protocols. *Haematologica*.

[16] Mulder T. A., Wahlin B. E., Österborg A., Palma M. (2019). Targeting the immune microenvironment in lymphomas of B-cell origin: from biology to clinical application. *Cancers*.

[17] Kluin P. M., Harris N. L., Stein H., Swerdlow S. H., Campo E., Harris N. L., Thiele J. (2008). B-cell lymphoma, unclassifiable, with features intermediate between large B-cell lymphoma and Burkitt lymphoma. *WHO Classification of Tumours of Haematopoietic and Lymphoid Tissues*.

[18] Younes S. F., Beck A. H., Lossos I. S., Levy R., Warnke R. A., Natkunam Y. (2010). Immunoarchitectural patterns in follicular lymphoma: efficacy of HGAL and LMO2 in the detection of the interfollicular and diffuse components. *The American Journal of Surgical Pathology*.

[19] Do B., Lossos I. S., Thorstenson Y., Oefner P. J., Levy R. (2003). Analysis of FAS (CD95) gene mutations in higher-grade transformation of follicle center lymphoma. *Leukemia and Lymphoma*.

[20] Armitage J. O., Longo D. L. (2016). Is watch and wait still acceptable for patients with low-grade follicular lymphoma. *Blood*.

[21] Batlevi C. L., Sha F., Alperovich A. (2020). Positron-emissiontomography-based staging reduces the prognostic impact of early disease progression in patients with follicular lymphoma. *European Journal of Cancer*.

[22] Thibaud V., Denève L., Dubruille S. (2021). Identifying frailty in clinically fit patients diagnosed with hematological malignancies using a simple clinico-biological screening tool: the HEMA-4 study. *Journal of Geriatric Oncology*.

[23] Mozas P., Rivero A., L´opez-Guillermo A. (2021). Past, present, and future of prognostic scores in follicular lymphoma. *Blood Reviews*.

[24] Mozas P., Rivero A., Rivas‐Delgado A. (2021). Prognostic ability of five clinical risk scores in follicular lymphoma: a single‐center evaluation. *Hematological Oncology*.

[25] Batlevi C. L., Sha F., Alperovich A. (2020). Follicular lymphoma in the modern era: survival, treatment outcomes, and identification of high-risk subgroups. *Blood Cancer Journal*.

[26] Barrington S. F., Mikhaeel N. G., Kostakoglu L. (2014). Role of imaging in the staging and response assessment of lymphoma: consensus of the international conference on malignant lymphomas imaging working group. *Journal of Clinical Oncology*.

[27] Rai A., Nastoupil L. J., Williams J. N. (2017). Patterns of use and survival outcomes of positron emission tomography for initial staging in elderly follicular lymphoma patients. *Leukemia and Lymphoma*.

[28] Abou-Nassar K. E., Vanderplas A., Friedberg J. W. (2013). Patterns of use of 18-fluoro-2-deoxy-D-glucose positron emission tomography for initial staging of grade 1-2 follicular lymphoma and its impact on initial treatment strategy in the National Comprehensive Cancer Network Non-Hodgkin Lymphoma Outcomes database. *Leukemia and Lymphoma*.

[29] Meignan M., Cottereau A. S., Versari A. (2016). Baseline metabolic tumor volume predicts outcome in high-tumor-burden follicular lymphoma: a pooled analysis of three multi-center studies. *Journal of Clinical Oncology*.

[30] Cottereau A. S., Versari A., Luminari S. (2018). Prognostic model for high-tumor-burden follicular lymphoma integrating baseline and end-induction PET: a LYSA/FIL study. *Blood*.

[31] Trotman J., Barrington S. F., Belada D. (2018). Prognostic value of end-of-induction PET response after first-line immunochemotherapy for follicular lymphoma (GALLIUM): secondary analysis of a randomised, phase 3 trial. *The Lancet Oncology*.

